# The complexity of skeletal fragility in overt endogenous Cushing’s syndrome: how much do we know?

**DOI:** 10.3389/fendo.2026.1918392

**Published:** 2026-10-29

**Authors:** Giacomo Voltan, Carlo Mogno, Giacomo Contini, Irene Tizianel, Michela Ferrarese, Valentina Camozzi

**Affiliations:** 1 Endocrine Unit, Department of Medicine (DIMED), Padua, Italy; 2 Endocrine Unit, University-Hospital of Padova, Padua, Italy

**Keywords:** bone quality, Cushing’s syndrome, endogenous hypercortisolism, fragility fractures, glucocorticoid-induced osteoporosis, skeletal fragility

## Abstract

Skeletal fragility is a major, yet still under-recognized, complication of endogenous Cushing’s syndrome, with a prevalence of densitometric osteoporosis of approximately 30-50% and fragility fractures reported in 11-76% of patients across published series. Chronic cortisol excess impairs bone strength through a complex network of direct and indirect mechanisms that collectively produce a deterioration of bone quality extending well beyond what is captured by standard densitometry. Therefore, fragility fractures occur in a substantial proportion of patients at bone mineral density values in the osteopenic or even normal range, underscoring the central role of bone microarchitectural impairment. Emerging tools, including the Trabecular Bone Score, high-resolution peripheral quantitative computed tomography, and bone marrow fat assessment by MRI, provide complementary information on bone quality and fracture risk. Following successful treatment of hypercortisolism, spontaneous skeletal recovery occurs but is slow, site-specific, and incomplete, with fracture risk remaining elevated for years after remission. Despite the clinical relevance of these complications, no CS-skeletal specific guidelines exist for their management, the evidence base for bone-active agents in endogenous CS is extremely limited, and real-world data confirm that bone health is systematically under-assessed in routine practice. This review provides a comprehensive and updated overview of the epidemiology, pathophysiology, risk assessment, and treatment of skeletal fragility in endogenous CS, with the aim of raising awareness and highlighting the urgent need for dedicated diagnostic and therapeutic strategies in this high-risk population.

## 1 Introduction

Cushing’s syndrome (CS) is a rare endocrine disorder caused by chronic endogenous cortisol excess, with an estimated annual incidence of roughly 0.7–2.4 new cases per million inhabitants per year (1). Most patients have ACTH-dependent CS, predominantly due to an ACTH-secreting pituitary adenoma, Cushing’s disease (CD), whereas ACTH-independent forms, including adrenal adenomas, carcinomas and bilateral adrenal hyperplasia, account for a minority of cases (2). Ectopic ACTH secretion from neuroendocrine tumors is less common but often presents with a more severe biochemical phenotype and a rapid onset of metabolic and systemic complications. Despite improvements in biochemical diagnostics and imaging techniques, diagnostic delay remains frequent because early clinical manifestations are non-specific and overlap with prevalent conditions such as simple obesity, metabolic syndrome, depression and polycystic ovary syndrome (3, 4).

Although CS is rare, its overall clinical burden is substantial, and it is associated with increased morbidity and premature mortality, mainly driven by cardiovascular and metabolic complications, including hypertension, diabetes, dyslipidemia and central obesity, as well as by thromboembolic and infectious events. Chronic hypercortisolism promotes a prothrombotic state, impairs immune function, induces profound changes in body composition and energy metabolism, and adversely affects multiple endocrine axes (5, 6), leading to sustained cardiometabolic risk even after cure in a subset of patients (7, 8). Within this complex clinical picture, skeletal fragility represents a major, yet still under-recognized, complication with significant impact on functional status, chronic pain, disability and quality of life (1, 9).

Bone complications, such as osteoporosis, osteopathy and fragility fractures, are frequent in patients with endogenous CS. Observational studies and cohorts from different geographic regions suggest that densitometric osteoporosis by dual-energy X-ray absorptiometry (DXA) may be present in approximately 30–50% of patients, with the lumbar spine being the most commonly involved site. Reported prevalence rates of fragility fractures vary widely, ranging from 11 to 76% (10–16). Vertebral fractures, often multiple, and rib fractures are the most frequently reported events, and might be the initial clinical manifestation (17).

Part of this variability reflects heterogeneity in the populations studied and the relatively small number of investigations specifically designed to evaluate skeletal outcomes in endogenous hypercortisolism. However, there is increasing consensus that the true fracture burden in CS is substantially underestimated. A major reason is that vertebral fragility fractures are often asymptomatic or only mildly symptomatic remaining thereby undetected unless actively sought by targeted imaging, such as lateral spine radiographs or vertebral fracture assessment (11, 16). Moreover, even in tertiary endocrine centers, systematic assessment of bone health may be overshadowed by the need to control overt cardiometabolic and infectious complications, resulting in suboptimal screening and follow-up of skeletal comorbidities.

Data from large registries and multicenter cohorts have provided important insights into real-world practice. Analyses showed that bone health is not consistently assessed in patients with CS across Europe, despite the high prevalence of skeletal comorbidities. Indeed, in a recent large analysis from the European Registry on Cushing’s Syndrome (ERCUSYN), it was reported that a DXA scan at diagnosis was performed in only 45% of patients and spinal radiographs in 29% of subjects (18).

The systematic screening is a crucial point, considering that DXA-based Bone Mineral Density (BMD) alone does not adequately reflect fracture risk in patients exposed to glucocorticoid excess. Hence, it’s widely known that in exogenous glucocorticoid-induced osteoporosis (GIO), fragility fractures frequently occur at BMD values only mildly reduced or even within the normal range (19, 20). Observational data in endogenous CS confirm these findings, showing that about 40% of patients with fragility fractures have BMD values in the osteopenic or normal range rather than in the osteoporotic one at the time of diagnosis (11, 21, 22). This pattern reflects the profound impact of glucocorticoid excess on the bone “quality,” including trabecular and cortical microarchitecture, bone geometry and material properties of the mineralized matrix and collagen, which are incompletely captured by standard DXA (23).

However, most of the available literature is derived from GIO in the context of exogenous CS, and although several mechanisms are shared, skeletal fragility in endogenous CS cannot be simply superimposed with classical exogenous GIO. First of all, in patients on long-term glucocorticoid therapy, the timing, dose and duration of exposure are usually known, allowing estimation of cumulative glucocorticoid burden and its temporal relationship with bone loss. By contrast, in endogenous CS the onset of hypercortisolism is insidious and often preceded by months to years of non-specific symptoms, so that bone damage accumulates silently before diagnosis. As a result, many patients with CS already harbor osteoporotic fractures at the time of diagnosis, frequently in the absence of prior skeletal symptoms (14, 24, 25).

Hence, the purpose of the present review is to provide an updated and comprehensive overview of skeletal fragility in endogenous overt CS, summarizing current evidence on its epidemiology, pathophysiology and clinical manifestations, and discussing available tools for risk assessment and treatment strategies.

## 2 Pathophysiological aspect of the bone damage in endogenous CS

### 2.1 Direct mechanisms of glucocorticoid-induced bone damage

The large body of evidence addressing the direct damage of glucocorticoids (GC) is derived mainly from the use of exogenous GC, since the exposure to supra-physiological levels of GC initiates a cascade of molecular events that systematically compromise the structural integrity of bone tissue through direct and coordinated mechanisms on resident cells. The impact on skeletal metabolism is profound, acting ubiquitously on all skeletal cell populations and their precursors through the glucocorticoid receptor (GR) (26–28). The damage is not limited to a simple reduction in bone mass, rather it extends to a systematic deterioration of the tissue’s architectural quality, which explains why fracture risk increases drastically and quickly, often occurring before significant changes in BMD (29–33). Bone imbalance is characterized by a biphasic actions of cortisol, which lead to a decoupling of bone turnover. Hypercortisolism during the initial phase of GC excess, causes a transient but rapid increase in bone resorption (34, 35). This is consequent to the enhanced secretion of receptor activator of nuclear factor kappa-B ligand (RANKL) and the drastic reduction of its decoy receptor, osteoprotegerin (OPG) (36–38). Consequently, the imbalance in the RANKL/OPG ratio dramatically accelerates the maturation and activity of osteoclasts (39, 40). Nevertheless, the core of the direct damage by hypercortisolism, lies in the suppression of the osteoblast lineage at every developmental stage. GC intervene at the level of mesenchymal stem cells in the bone marrow, forcing a shift of the differentiating from active osteoblasts toward the adipocytic lineage (26, 38, 41, 42). This lineage-specific “switch” is driven by the induction of pro-adipogenic transcription factors, such as peroxisome proliferator-activated receptor gamma 2 (PPARgamma2) and CCAAT/enhancer binding protein alpha (C/EBPa) which is accompanied by the simultaneous downregulation of Runx2, the essential transcription factor for osteogenesis (43–46). This phenomenon is clinically manifested as an increase in bone marrow adiposity that correlates inversely with bone mass loss. Beyond reducing the pool of available precursors, glucocorticoids paralyze the proliferation and maturation of committed osteoblasts. They induce cell cycle arrest in the G1 phase by downregulating activators like Cyclin A and D and cyclin-dependent kinases (CDK2, 4, 6), while promoting the expression of inhibitors such as p21, p27, and p53 (47–49).

This proliferative blockade is further exacerbated by direct interference with primary bone-anabolic signaling pathways, specifically the Wnt system and bone morphogenetic proteins (BMPs). GC significantly increase the production of Wnt antagonists, including sclerostin (SOST) and Dickkopf-1 (DKK1), by both osteoblasts and osteocytes (50–54). These inhibitors prevent the binding of Wnt ligands to their co-receptors (LRP5/6), leading to the degradation of beta-catenin and suppressing the expression of genes required for synthetic function, including type I collagen and osteocalcin (27, 55, 56).

An equally critical and often underestimated target is the osteocyte. These cells, representing over 95% of the total bone cell population, form a dense communicating network within the mineralized matrix and act as the guardians of bone quality and mechanotransduction (57, 58). Excess GC compromise their function and survival in a time- and dose-dependent manner, triggering apoptosis and leading to the degeneration of the lacuno-canalicular network (59–61). This architectural decay compromises the structural integrity of the bone by impairing the perilacunar environment and increasing the tissue fragility, regardless of the remaining mineral mass (62–64).

While the first phase of the bone damage encompasses a prevalent high turnover state, the advanced phase of damage evolves toward a low-turnover state, concomitant to the osteocytes and osteoblasts impairment, where both formation and resorption are suppressed (65, 66).

Vertebral fractures, frequently multiple, and rib fractures are the most commonly reported skeletal events in endogenous CS. This anatomical distribution is biologically plausible because glucocorticoid excess preferentially affects trabecular-rich skeletal compartments, including the vertebrae and ribs. The high remodeling surface of trabecular bone amplifies the consequences of suppressed osteoblast activity, osteocyte dysfunction, and loss of trabecular connectivity, thereby reducing resistance to mechanical loading (67).

### 2.2 Indirect systemic mechanism contributing to bone fragility

Beyond the direct cellular effects of GC excess on the bone cells, endogenous hypercortisolism carries several systemic alterations that might substantially amplify the skeletal fragility.

Chronic cortisol excess exerts an inhibitory effect on hypothalamic GnRH secretion and pituitary gonadotropin release, leading to functional hypogonadotropic hypogonadism in a large proportion of patients with active CS (68, 69). This mechanism is particularly relevant in younger men and premenopausal women (70, 71), in whom abrupt sex steroid deprivation superimposes on glucocorticoid-mediated damages. Interestingly, despite sex hormones defects directly impact the BMD (72), some reports suggest that gonadal status might influence the risk of fractures especially in patients with a mild increase in cortisol secretion, whereas in patients with overt CS the potential protective effect of eugonadism is overcome by overt hypercortisolism (15).

Endogenous CS also impairs the growth hormone (GH)/insulin-like growth factor 1 (IGF-1) axis, inhibiting GH secretion, and hampering the IGF-1 action, thus promoting a state of functional GH deficiency (GHD) with loss of anabolic input to both bone and muscle (73, 74). In a recent study enrolling patients with CD, lower IGF-1 levels have been independently associated with low bone mass, whereas the patients with the highest tertile of the IGF-1 levels had a lower prevalence of low bone mass (75). Both GHD and chronic hypercortisolism lead to a low bone turnover osteoporosis, potentially and sinergically increasing the risk of fracture (76, 77). The idea that GHD and endogenous hypercortisolism might converge versus a common bone damage is reinforced by the findings that patients with GHD and a history of CD in remission experienced a greater increase in lumbar BMD after two years of GH replacement therapy compared with GHD patients without previous CD (78).

Alterations in calcium and phosphate metabolism represent another important indirect mechanism of skeletal damage. Indeed, endogenous and exogenous GC hamper intestinal calcium absorption, while simultaneously leading to an increased urinary calcium excretion, resulting in a chronic negative calcium balance (79). Recently, alterations in phosphate metabolism have been reported in active CD, with hypophosphatemia occurring more commonly in affected patients than in healthy controls and improving after successful treatment of hypercortisolism (80). A potential underlying mechanism may involve FGF23-induced phosphate wasting, which has been observed during exogenous glucocorticoid treatment (81). Disruption in PTH homeostasis, should also be reminded in CS context. Interestingly, changes in PTH secretion, with evidence from GIO, have been described showing an increase in pulsatile PTH release accompanied by a reduction in tonic PTH secretion (82).

Importantly, the vitamin D metabolism in hypercortisolism appears deeply impaired, thus, despite scant data, patients with endogenous CS usually show lower 25-hydroxyvitamin D (25OHD) than controls (83, 84). Proposed mechanisms, mainly deriving from the use of exogenous GC, include increased hepatic and renal catabolism of vitamin D metabolites, downregulation of vitamin D receptor (VDR) expression in target tissues, and GC-mediated suppression of renal 1α-hydroxylase, the enzyme responsible for converting 25(OH)D to its active form (85–87). In addition, fat-soluble vitamin D might be sequestrated in adipose tissue, limiting its bioavailability in obese individuals (88, 89). This “trapping” of vitamin D by adipose tissue, seems to be supported also by the fact that higher doses of cholecalciferol are needed in the supplementation of obese individuals (90).

If we keep in mind that altered fat distribution and visceral obesity are pivotal hallmarks of CS, the disruption of vitamin D metabolism may be emphasized.

Taken together, these observations indicate that endogenous hypercortisolism induces a potential harmful effect on the PTH-vitamin D and calcium-phosphate axis, which acts as an additional amplifier of bone fragility, beyond the direct effect of hypercortisolim.

Sarcopenia and myopathy impair muscle function and muscle–bone crosstalk, both mechanically and through endocrine/paracrine pathways, thereby increasing the likelihood of falls and fragility fractures (91, 92). Consistently, the CS-associated myopathy is a central feature of the musculoskeletal phenotype and a key indirect contributor to fracture risk. Patients typically present with painless proximal muscle weakness, particularly in the lower limbs, impaired chair-rise and stair-climbing ability, reduced physical performance, while histological and imaging data show muscle atrophy and increased intramuscular fat infiltration (93). In dedicated series, objective muscle dysfunction is detected in most patients with active CS and may persist for years after biochemical remission, despite normalization of cortisol levels (94, 95). Mechanistically, hypercortisolism up-regulates proteolytic pathways and down-regulates IGF-1-mediated anabolic signaling (96).

The broader sarcopenia literature consistently reports strong associations between low muscle mass or function, and both falls and fragility fractures, supporting the concept of a coupled “osteosarcopenic” phenotype (97). Hence, from an integrate point of view, the CS-myopathy contribution to the overall skeletal fragility should be particularly kept in mind.

Finally, metabolic comorbidities represent additional and largely independent contributors to skeletal fragility. Growing evidence indicates that central obesity, insulin resistance, and diabetes mellitus adversely affect bone health through several mechanisms, including chronic low-grade inflammation, increased bone marrow adiposity, dysregulated adipokine and myokine secretion, reduced physical activity, and the accumulation of advanced glycation end products within bone collagen (98–100). Notably, these metabolic abnormalities are hallmark features of Cushing’s syndrome and may therefore further exacerbate the detrimental effects of hypercortisolism on the skeleton, further reinforcing the idea that skeletal fragility in endogenous CS should be understood as the result of a disease-specific network of endocrine, muscular, and metabolic alterations, rather than being equated with “classic” glucocorticoid-induced osteoporosis in patients on exogenous steroids. The main mechanisms of bone damage are resumed in **Figure 1**.

**Figure 1:**
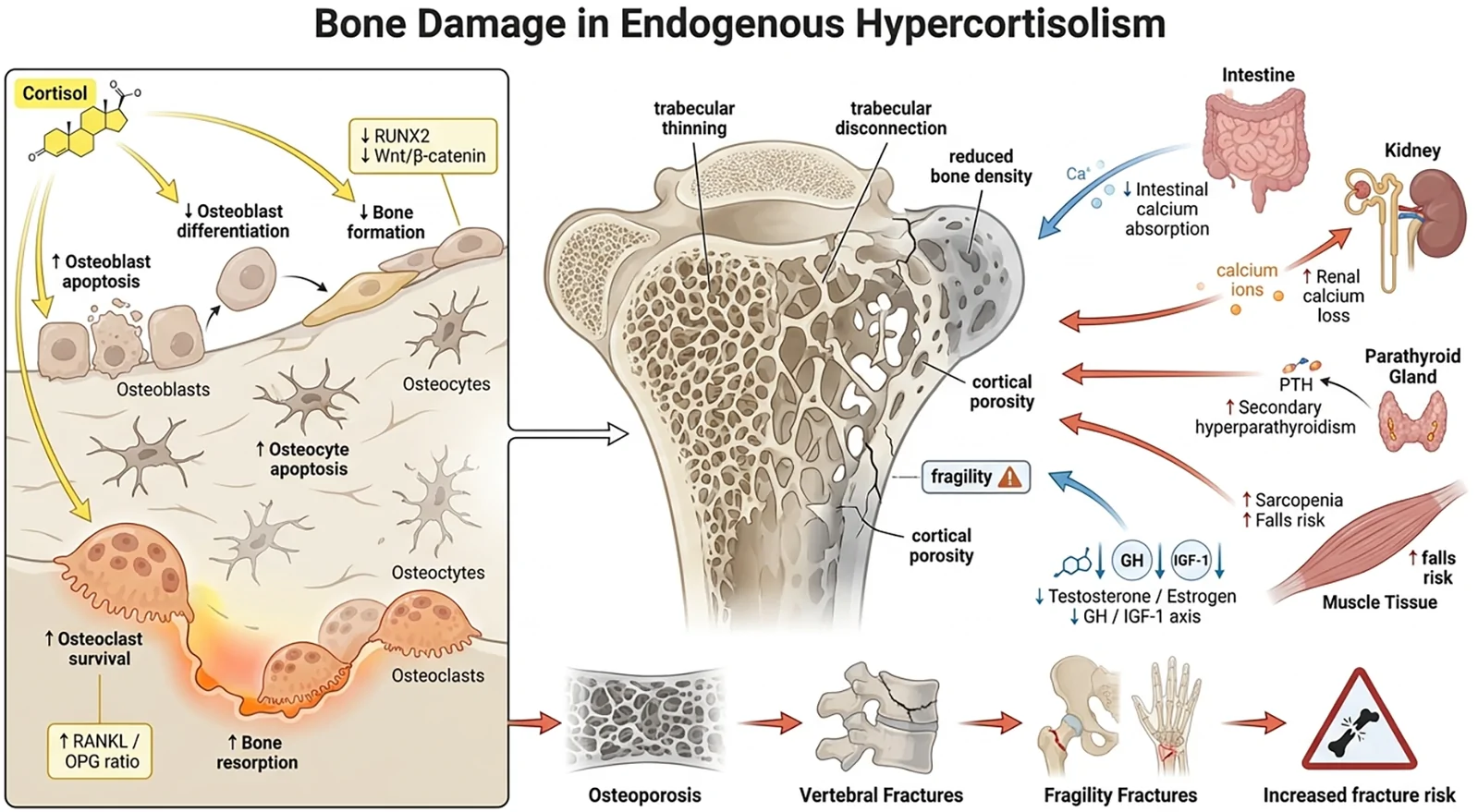
Bone damage in endogenous hypercortisolism. The schematic illustrates how chronic cortisol excess impairs bone strength through integrated direct and indirect mechanisms at the cellular, skeletal and systemic level. On the left, cortisol suppresses osteoblast differentiation and function via downregulation of RUNX2 and Wnt/β-catenin signaling, increases osteoblast apoptosis, induces osteocyte apoptosis, and enhances osteoclast survival by increasing the RANKL/OPG ratio, leading to accelerated bone resorption and subsequent low-turnover bone loss. In the central panel, these changes translate into trabecular thinning and disconnection, cortical porosity and reduced bone density, ultimately resulting in osteoporosis, vertebral fractures, peripheral fragility fractures and a marked increase in overall fracture risk. On the right, systemic effects contribute further to skeletal fragility: reduced intestinal calcium absorption and increased renal calcium loss promote secondary hyperparathyroidism; alterations of sex steroids and the GH/IGF-1 axis impair bone formation and muscle mass; sarcopenia and muscle weakness increase falls risk, thereby amplifying fracture risk in patients with endogenous hypercortisolism. GH, growth hormone; IGF-1, insulin-like growth factor 1; OPG, osteoprotegerin; PTH, parathyroid hormone; RANKL, receptor activator of nuclear factor-κB ligand; RUNX2, runt-related transcription factor 2.

## 3 Instrumental and biochemical assessment of bone impairment in Cushing’s syndrome

In clinical practice, BMD assessment at the lumbar spine, hip and femoral neck by DXA, based on World Health Organization (WHO) criteria, represents the first level exam for the diagnosis of primary and secondary osteoporosis (101). In the context of CS fragility fractures typically occur at higher BMD than those observed in primary osteoporosis (56). As previously described, bone quality impairment, which involves bone geometry, macro- and microarchitecture, porosity, mineralization, and matrix composition, represents the main cause of bone fragility in these patients, rather than low mineral density (21). As BMD is an indicator of bone quantity, and DXA analysis does not provide direct information on bone quality (102), this parameter is not sufficient to predict fracture risk in Cushing’s syndrome. Therefore, although a BMD measurement is recommended, the recent consensus on diagnosis and management of Cushing’s disease also suggests that a bone quality assessment or vertebral morphometry should be performed in all patients (103). New instruments to evaluate bone quality are emerging, including quantitative ultrasonometry (QUS), quantitative computed tomography (QCT), magnetic resonance imaging (MRI), and trabecular bone score (TBS) measurement by DXA (104). Specifically, TBS, is derived from a gray-level texture analysis of two-dimensional lumbar spine images acquired by DXA and represents a quantitative indicator of bone microarchitectural status (105). A low TBS score indicates impaired bone quality, characterized by reduced trabecular number and thickness (106). In patients with CS, the majority of the available studies are concordant in identifying reduced TBS values, confirming the deep impairment in the bone quality (107–109). Notably, an inverse correlation between this parameter and the duration and severity of CS has been described, but especially between the number and severity of vertebral fractures, regardless of BMD (110–113). Hence, despite some author did not confirm it as an independent predictor of fractures (109, 112), TBS may represent a more accurate parameter than BMD for assessing fracture risk in CS.

Among the most recently developed DXA-derived tools, the Bone Strain Index (BSI), a parameter based on finite element analysis (FEA) of DXA images that integrates bone mineral density distribution, bone geometry, and loading conditions, has been shown to predict fragility fracture risk independently of BMD across several primary and secondary osteoporosis settings, including primary hyperparathyroidism. Although no specific data are currently available in Cushing’s syndrome, the rationale for its application is supported both by the dissociation between BMD and fracture risk characteristic of hypercortisolism and by the fact that it provides a direct estimate of bone tissue mechanical strain under physiological loading conditions, thereby potentially capturing structural strength impairment that is not detected by conventional densitometry (114).

Alternative options for evaluating bone microarchitecture include quantitative computer tomography (QCT) and high-resolution peripheral quantitative computed tomography (HR-pQCT). These methods enable measurement of cortical thickness and porosity, trabecular number, thickness and separation, and trabecular bone volume fraction (21, 102). A few studies performing QCT (115) and HR-pQCT (116) on patients with CS confirmed the presence of reduced vertebral trabecular volumetric BMD and impaired cortical bone microarchitecture. However, these imaging techniques are not applicable as screening tools in clinical practice, due to the high costs, the radiation dose, and their limited availability (117).

Recently, a correlation has emerged between bone marrow adiposity, assessed with MRI, and the prevalence of vertebral fractures (118). In patients affected with Cushing’s syndrome, Ferraù et al, described higher bone marrow fat (BMF) and vertebral fractures rates compared to controls, unrelated to BMD values (119). An inverse correlation between BMF and TBS scores was also described (120). Therefore, MRI may represent a further instrument for obtaining a surrogate evaluation of the bone quality in CS.

Given the high, though underestimated, prevalence of vertebral fractures in Cushing’s syndrome, and their precocious and often asymptomatic occurrence, an early morphometric assessment is essential in the diagnostic work-up (21, 56). Vertebral morphometric evaluation is usually performed on X-ray lateral images of the spine, but it is also feasible using DXA-based Vertebral Fracture Assessment (VFA) (104). Vertebral fractures should be identified and classified according to the Genant semi-quantitative method based on the quantification of anterior, middle, and posterior vertebral body heights (121). Due to the low cost and radiation dose, this exam should be recommended in all patients with Cushing’s syndrome at diagnosis (46, 102).

With regard to biochemical parameters, although several studies have shown abnormal bone turnover markers levels, reflecting increased bone resorption and suppressed bone formation, their role in bone health assessment in Cushing’s syndrome is still a matter of debate (122–124). Several studies have demonstrated a negative correlation between osteocalcin levels and cortisol concentrations (11, 107, 110), whereas findings regarding the type I collagen C-terminal telopeptide (CTX-I), a marker of bone resorption, have been inconsistent, with studies reporting either increased or decreased levels in patients with hypercortisolism compared with healthy controls. This discrepancy may reflect a time-dependent relationship, characterized by an early increase in bone resorption during the initial phase of hypercortisolism, followed by a subsequent decline occurring in parallel with the persistent suppression of bone formation (46). Supporting this hypothesis, a cohort study including 89 patients with Cushing’s syndrome showed a three- to fourfold increase in bone formation markers during the first year after surgery, followed by a more moderate decline thereafter, highlighting the importance of interpreting biochemical markers within the temporal context of disease activity and recovery (125).

## 4 Clinical predictors of fracture risk in Cushing’s syndrome

As previously discussed, although in the context of CS, fragility fractures frequently occur in the presence of normal or slightly reduced BMD (12, 13, 17), a correlation between lower lumbar spine BMD and a higher fracture risk has been demonstrated (11, 22). However, BMD alone appears insufficient to adequately predict fracture risk, hence, an integrated approach, including the identification of clinical predictors, is pivotal for a further stratification.

In this regard, it’s important to bear in mind that there are currently no validated algorithms for fracture risk prediction in patients with endogenous hypercortisolism. Consistently, the Fracture Risk Assessment Tool (FRAX) may not be applicable in this context, as it’s not possible to accurately quantify cortisol exposure, differently from exogenous hypercortisolism (126, 127).

As well as other primary and secondary osteoporosis, previous fractures represent the strongest predictor of bone fragility, thus, affected patients have a significantly higher probability of experiencing new fractures during follow-up (14).

Among clinical features, gender differences in fracture risk are well documented in the literature. In line with higher rate of metabolic and cardiovascular complications (128) male patients with CS seem to be affected by a deeper bone fragility, exhibiting higher prevalence of spine and hip fractures (12, 18, 107, 109). This subgroup of patients is also characterized by lower lumbar spine BMD (12, 129, 130).

Other clinical predictors of fractures, are represented by age at diagnosis (16, 131) and BMI (128, 130). The severity and duration of disease also appear to be predictors of fracture risk, with a positive correlation between these variables and fracture rate (11, 13, 16, 109). Interestingly, van Houten et al. showed a correlation between a shorter duration of symptoms and lower BMD levels at diagnosis, however, this could be explained by a longer, more insidious course of the disease prior to diagnosis (130).

A possible indicator of disease severity at diagnosis may be represented by the degree of hypercortisolism. Notably, the association between cortisol hypersecretion and the risk for vertebral fracture, independent of BMD levels, has been confirmed by several studies. A few studies addressing Mild Autonomous Cortisol Secretion (MACS) found a correlation between higher serum cortisol levels after a 1 mg overnight dexamethasone-suppression test (DST) and the development of new vertebral fractures during follow-up (132–134), even if the description of bone fragility in MACS is beyond the purpose of this review. Morning cortisol serum levels have also been shown to be predictive of vertebral fractures by Sun et al. (135) but this result has not been confirmed by other studies (16, 22). Similarly, 24h urinary free cortisol (UFC) was demonstrated to be a predictive parameter in some studies (13, 107) whereas other authors did not find a significant association with BMD or fracture risk (22, 130, 136, 137).

The possible predictive role of the CS etiology is controversial. Several studies have shown that bone involvement may be more severe in ACTH-independent than in ACTH-dependent CS (10, 136). This discrepancy is biologically plausible, and seems to be related to the protective effect of adrenal androgens, particularly DHEAS, on bone integrity. However, more recent studies did not confirm this evidence (12, 14, 18, 22, 130), hypothesizing that the chronic bone damage exerted by cortisol excess, might largely overcome the beneficial effects of DHEAS.

Patients with ectopic ACTH syndrome may nevertheless present with particularly severe skeletal involvement. In a prospective study of 80 patients with endogenous CS, the 10 patients with ectopic ACTH secretion had the lowest lumbar BMD and a higher prevalence of multiple vertebral fractures than patients with adrenal CS. However, the prevalence of any vertebral or clinically apparent fracture did not differ significantly across etiological groups (11). A subsequent small retrospective cohort similarly reported a numerically higher fracture prevalence in ectopic CS than in pituitary or adrenal CS, but without statistically significant differences in either fractures or densitometric osteoporosis (138). Therefore, the available evidence suggests a potentially more severe skeletal presentation in ectopic ACTH syndrome, but does not establish a distinct etiology-specific bone phenotype independent of the greater intensity and rapid progression of hypercortisolism.

Finally, a recent study by our group identified a correlation between fracture risk and the presence of atherosclerotic plaques suggesting that cardiovascular morbidity may represent a further predictor of bone impairment in Cushing’s syndrome (109). Clinical predictors are resumed in **Table 1**.

**Table 1:** Clinical predictors of fracture risk in endogenous Cushing’s syndrome.

Factor	Type of association	References
Lumbar spine BMD	Correlation between lower BMD and higher fracture risk.	Tauchmanovà et al. (2006) (11) Apaydın et al. (2021) (22)
Previous fractures	Strongest predictor of bone fragility; higher probability of new fractures.	Vestergaard et al. (2002) (14)
Male Sex	Associated with deeper bone fragility, higher prevalence of spine/hip fractures, and lower lumbar BMD.	Valassi et al. (2011) (12) Zdrojowy-Wełna et al. (2026) (18) Belaya et al. (2015) (107) Voltan et al. (2025) (109) Broersen et al. (2019) (129) Van Houten et al. (2021) (130)
Age at diagnosis	Higher rate of fractures in elderly CS patients	Füto et al. (2008) (16) Mancini et al. (2004) (131)
BMI	Identified as a clinical predictor of fractures.	Zilio et al. (2014) (128) Van Houten et al. (2021) (130)
Disease severity and duration	Positive correlation between these variables and fracture rate.	Tauchmanovà et al. (2006) (11) Trementino et al. (2014) (13) Füto et al. (2008) (16) Voltan et al. (2025) (109)
Degree of hypercortisolism	Morning cortisol levels were predictive of vertebral fractures in some studies; not confirmed by others. 24h-UFC was predictive of fractures in some studies, but without significant association in others.	Sun et al. (2023) (135) Füto et al. (2008) (16) Apaydın et al. (2021) (22) Trementino et al. (2014) (13) Belaya et al. (2015) (107) Apaydın et al. (2021) (22) Van Houten et al. (2021) (130) Minetto et al. (2004) (136) Randazzo et al. (2012) (137)
CS Etiology	Controversial: possibly more severe in ACTH-independent CS, but not confirmed by recent studies.	Ohmori et al. (2003) (10) Minetto et al. (2004) (136) Valassi et al. (2011) (12) Vestergaard et al. (2002) (14) Zdrojowy-Wełna et al. (2026) (18) Apaydın et al. (2021) (22) Van Houten et al. (2021) (130)
Cardiovascular morbidity	Correlation between fracture risk and the presence of atherosclerotic plaques.	Voltan et al. (2025) (109)

BMD, bone mineral density; BMI, body mass index; CS, Cushing’s syndrome; UFC, urinary free cortisol.

## 5 Bone recovery after CS remission

As many other secondary osteoporosis (139, 140), the first line treatment of CS-related skeletal fragility is the correction of cortisol excess (103), thereby eliminating the underlying etiological factor. Successful control of hypercortisolism is directly associated with improvements in bone health.

Notably, upon achieving biochemical remission, a spontaneous “bone healing” phenomenon is triggered, characterized by a marked surge in bone turnover. During this phase, bone formation marker, such as osteocalcin and procollagen type I amino-terminal propeptide (PINP), significantly outpace the rise in bone resorption markers like CTX, thereby establishing a net positive balance for mineralization and restoring osteoformative capacity. This process begins as early as the first 3–6 months post-remission, reflecting a post-remission “anabolic window” driven by the release of osteoblast-related genes from chronic glucocorticoid inhibition, and subsequently tapering off during the second year. A recent German prospective study involving 89 patients with Cushing’s Syndrome (CS) documented that lumbar T-score improved in 78% of subjects two years after surgical cure, confirming that the immediate post-remission period is marked by substantial spontaneous bone recovery (125). Although the gain in BMD is most pronounced within the first 2–5 years, the overall improvement appears to be an extremely slow and progressive process. A Dutch cohort study of 231 patients demonstrated that lumbar Z-score improvements remained significant even 20 years after remission, with men exhibiting a more robust recovery than women (130). Furthermore, BMD recovery is known to be site-specific, being more rapid and pronounced at the lumbar spine than the femoral neck, likely due to the higher metabolic sensitivity of trabecular bone to glucocorticoid-induced damage (16, 125, 130, 141, 142). A small retrospective study reported that BMD recovery is more appreciable in patients successfully managed surgically compared to those receiving medical therapy with steroidogenesis inhibitors (143). These findings should therefore not be interpreted as evidence of an intrinsic bone-specific superiority of surgery; rather, they may reflect differences in patient selection and in the rapidity, completeness, and durability of cortisol normalization. Importantly, recovery of areal BMD should not be considered synonymous with complete restoration of skeletal strength. Different components of bone competence may recover at different rates, and persistent abnormalities may remain undetected by conventional DXA. Hence, to fully characterize skeletal health following hypercortisolism cure, it is essential to evaluate qualitative bone parameters along with densitometric data. These qualitative markers appear to normalize earlier and more significantly than quantitative ones, as shown in a recent study where improvements in the TBS preceded and exceeded those in BMD (144). Despite these improvements, alterations in mechanical resistance may persist many years after cure. A recent study evaluated biomechanical properties in 32 women in long-term CS remission using FEA based on proximal femur QCT images, simulating a sideways fall to measure mechanical stress and strain. Even when BMD and TBS values were comparable to healthy controls, all FEA-derived biomechanical parameters were significantly worse in subjects with prior hypercortisolism, suggesting a persistent impairment of skeletal strength and a localized increase in fracture risk (145). Consistently, a recent impact microindentation study showed a lower Bone Material Strength Index in patients with previous CS than in age-, sex-, and BMD-matched controls (146).

These findings suggest that geometric, biomechanical, and bone material abnormalities may represent a persistent skeletal footprint of previous cortisol excess and may contribute to residual fracture susceptibility after biochemical remission.

Persistent fracture susceptibility after remission may also involve extra-skeletal factors. Indeed, long-term studies have reported persistent muscle weakness, increased thigh muscle fat infiltration, and impaired gait, balance, and chair-rise performance in patients considered biochemically cured. Moreover, abdominal adiposity, diabetes, dyslipidaemia, and other cardiometabolic abnormalities may improve only partially after remission (8, 93, 94) and could further impair the muscle-bone unit and increase fall propensity. However, their direct contribution to incident fractures after CS remission has not yet been quantified, and these factors should therefore be regarded as plausible contributors rather than established mediators of residual fracture risk.

Postoperative glucocorticoid replacement should also be considered when interpreting skeletal recovery after CS remission. Replacement therapy is essential in patients with postoperative hypocortisolism (103, 147); however, prolonged exposure to supraphysiological doses may partially perpetuate glucocorticoid-mediated suppression of bone formation and attenuate the post-remission anabolic response. In women with long-term remission of CS, the duration of glucocorticoid replacement was negatively associated with lumbar BMD and bone mineral content and emerged as a significant predictor of impaired bone mass in multivariable analysis (148). Therefore, postoperative management should aim for the lowest effective replacement dose, with regular clinical and biochemical reassessment of hypothalamic-pituitary-adrenal axis recovery and discontinuation when normal adrenal function has been documented, while avoiding both adrenal insufficiency and unnecessarily prolonged over-replacement.

Consequently, the issue of residual fracture risk remains highly relevant.

In a Swedish nationwide longitudinal study of 419 patients with a median remission duration of 13 years, the standardized incidence ratio for fractures remained 1.7 times higher than that of the general population (149). Recently, Behary and colleagues, in a cohort of 79 CS patients, described a fractures rate of 17.7% within 2 years of the diagnosis, whereas 12 additional fractures were reported during the follow-up, despite CS remission (150). Therefore, while endocrine remission is the fundamental prerequisite for skeletal recovery, it does not necessarily coincide with a complete normalization of fracture risk. Notably, this strictly mirrors the persistence of cardiovascular risk after CS remission, which might lead to an overall increased mortality even after several year after the correction of hypercortisolism (6, 7). Nonetheless, evidence regarding fracture-risk normalization after remission remains scant and heterogeneous, since several differences in study design, sample size, follow-up duration, fracture ascertainment, and the inclusion or exclusion of asymptomatic vertebral fractures may partly explain these discrepancies (6, 14, 151).

## 6 Treatment of bone fragility in CS

As previously mentioned, the definitive treatment of hypercortisolism represents the single most effective measure for restoring skeletal health in endogenous CS and constitutes the absolute therapeutic priority. No specific evidence-based guidelines exist for the management of osteoporosis in endogenous CS, and current guidance merely suggests that additional anti-osteoporotic medication may be beneficial in accelerating BMD recovery or preventing further bone loss in patients not in remission (103). Recommendations developed for GIO (152) cannot be automatically translated to endogenous CS for several reasons: there is no validated method to convert endogenous cortisol excess into a glucocorticoid-equivalent dose for FRAX-based risk stratification; the pathophysiological milieu of CS is substantially more complex, and the natural history of skeletal disease is profoundly shaped by the diagnostic delay. Hence, a particularly important difference concerns the timing of bone resorption: in exogenous GIO the initial phase of enhanced resorption defines the window of greatest antiresorptive efficacy, whereas in endogenous CS this phase is frequently missed at diagnosis; moreover, in patients undergoing curative surgery, antiresorptive therapy may theoretically interfere with the physiological recovery in bone turnover that underlies post-remission bone healing (24). Several general recommendations from GIO management can nonetheless be applied to all patients with CS. Adequate calcium intake (1000–1200 mg/day from dietary and supplemental sources) and vitamin D supplementation targeting serum 25(OH)D levels above 30 ng/mL should be recommended. Smoking cessation, reduction of alcohol consumption, and structured exercise programs emphasizing resistance and balance training are equally important, particularly given the increased risk of falls attributable to glucocorticoid-induced myopathy (152, 153). Among pharmacological interventions, bisphosphonates are the most widely prescribed bone-active agents in endogenous CS in clinical practice, as confirmed by ERCUSYN registry data (18). In the context of GIO bisphosphonates largely demonstrated to reduce the risk of vertebral fractures (154), however the evidence in endogenous hypercortisolism are limited. The only randomized trial in this population comprising 39 patients with Cushing’s disease treated with alendronate in combination with ketoconazole demonstrated a significantly greater improvement in lumbar spine and femoral neck BMD at 12 months compared with placebo (155). Conversely, a recent retrospective study comparing bisphosphonate-treated and untreated patients following surgical remission found no significant difference in BMD recovery at 12 months despite greater suppression of bone turnover markers in the treated group (156). Notably, this might be linked to the greater efficacy of the antiresorptive therapy when the osteoclast activity is prevalent. Denosumab has robust efficacy in GIO, with vertebral fracture reduction superior to oral bisphosphonates in comparative meta-analyses (157). Its rapidly reversible mechanism of action makes it theoretically attractive in patients with CS awaiting curative surgery, as it avoids skeletal accumulation (158). However, no controlled data exist in endogenous CS, and the well-documented rebound risk upon discontinuation characterized by a sharp surge in bone turnover markers and a risk of multiple vertebral fractures occurring demands careful planning, especially considering the high bone turnover state following CS remission. Anabolic therapies have the strongest pathophysiological rationale in endogenous CS, given that the prevalent underlying skeletal defect is suppressed bone formation rather than the initial rapid phase of excess resorption (19, 21, 46). Teriparatide is the only anabolic agent approved for GIO, however, in endogenous CS, evidence is limited to isolated case reports documenting significant BMD gains (159, 160).

Romosozumab, a dual-action sclerostin inhibitor, is theoretically intriguing given the role of Wnt pathway suppression in glucocorticoid-induced bone disease. However, evidence on circulating sclerostin levels in endogenous CS are contradictory (161, 162) and no data on romosozumab efficacy or safety are available in endogenous CS.

## 7 Unmet needs and clinical gaps

Despite the clinical relevance of skeletal complications in CS, their assessment and management remain suboptimal in routine practice. In the recent ERCUSYN analysis (18), osteoporosis and fractures were common at diagnosis, thus DXA was performed in only about half of patients, and vertebral imaging was obtained far less consistently. This strongly suggests that occult vertebral fractures are still underdetected. A major gap is that skeletal evaluation in CS still too often stops at BMD measurement, although bone quality impairment plays a central role in glucocorticoid excess and may explain fractures occurring at non-osteoporotic BMD values (19, 46, 140). In this context, tools beyond standard DXA, such as TBS and advanced imaging of bone microarchitecture, despite being insufficiently integrated into dedicated care pathways for CS, they have been recently suggested in the diagnostic work-up (117). **Figure 2** summarizes a proposal holistic management for bone fragility in CS.

**Figure 2:**
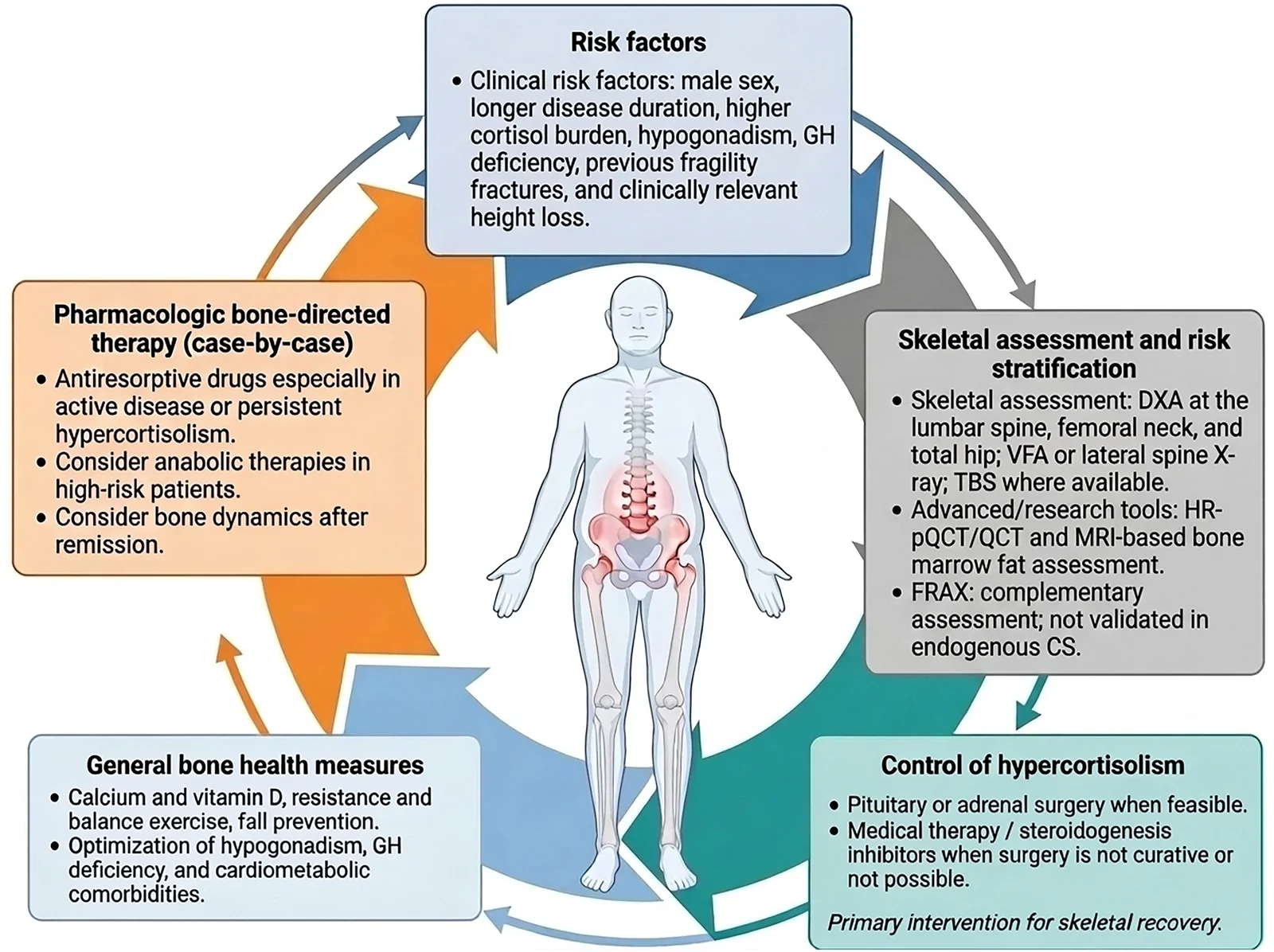
Patient-centered conceptual pathway for the assessment and management of skeletal fragility in endogenous Cushing’s syndrome. The pathway begins with the identification of major clinical risk factors, including male sex, longer disease duration, higher cortisol burden, hypogonadism, growth hormone deficiency, previous fragility fractures, and clinically relevant height loss. Routine skeletal assessment should integrate DXA at the lumbar spine, femoral neck, and total hip; vertebral fracture assessment or lateral spine radiography; and trabecular bone score, where available. FRAX may be used as a complementary assessment tool, although it has not been validated in endogenous Cushing’s syndrome and should not replace disease-specific clinical judgment. Advanced approaches, including HR-pQCT, QCT, and MRI-based bone marrow fat assessment, may provide additional information on bone microarchitecture, volumetric density, biomechanical competence, and marrow adiposity; however, these techniques remain largely confined to research or specialized settings. Control of hypercortisolism represents the primary intervention for skeletal recovery. General bone-health measures and pharmacological bone-directed therapy should be tailored to disease activity, fracture history, skeletal assessment, comorbidities, and bone dynamics following remission. CS, Cushing’s syndrome; DXA, dual-energy X-ray absorptiometry; FRAX, Fracture Risk Assessment Tool; GH, growth hormone; HR-pQCT, high-resolution peripheral quantitative computed tomography; MRI, magnetic resonance imaging; QCT, quantitative computed tomography; TBS, trabecular bone score; VFA, vertebral fracture assessment.

Further unmet needs concern treatment strategy and access to therapy. There is still no CS-specific algorithm integrating fracture history, disease severity, duration of hypercortisolism, and indices of bone quality to guide risk stratification and treatment selection, and clinicians largely rely on evidence extrapolated from primary osteoporosis or glucocorticoid-induced osteoporosis (152). This issue is particularly relevant when considering therapeutic strategies, as the optimal timing, sequencing, and duration of antiresorptive and osteoanabolic treatments, throughout the various phases of the disease, remain poorly defined. Consequently, clinical decisions are frequently guided by extrapolation from other forms of secondary osteoporosis rather than by disease-specific evidence. Furthermore, despite the markedly elevated fracture risk observed in patients with endogenous CS, important regulatory barriers continue to hinder the translation of pathophysiological knowledge into clinical practice. A notable example is provided by current Italian reimbursement criteria for osteoanabolic therapies (163): teriparatide is reimbursable for patients with glucocorticoid-induced osteoporosis carrying 1 fracture, whereas endogenous hypercortisolism is not explicitly included among the eligible conditions. This discrepancy creates a significant divergence between clinical fracture risk and access to appropriate treatment.

Altogether, these gaps underscore the need for prospective studies specifically designed to address bone complications in endogenous CS and to define standardized diagnostic pathways, establishing tailored therapeutic algorithms that better reflect the specific skeletal burden of endogenous CS.

## 8 Conclusions

Skeletal fragility is a major and still under-recognized complication of endogenous Cushing’s syndrome. Chronic hypercortisolism impairs bone strength through multiple mechanisms that extend beyond bone mineral density, resulting in a markedly increased fracture risk that may persist even after disease remission. Current evidence supports considering endogenous CS as a condition of very high fracture risk, warranting early assessment and proactive management of bone health. Future studies are needed to better define optimal therapeutic strategies, while updated clinical and regulatory frameworks should ensure that treatment access adequately reflects the true impact of skeletal disease in this population.
